# Advancing precision livestock farming: integrating artificial intelligence and emerging technologies for sustainable livestock management

**DOI:** 10.5713/ab.25.0289

**Published:** 2025-08-12

**Authors:** L. O. Tedeschi, Pablo Guarnido-Lopez, Hector M. Menendez, Seongwon Seo

**Affiliations:** 1 Department of Animal Science, Texas A&M University, College Station, TX, USA; 2 Department of Animal Science, South Dakota State University, Rapid City, SD, USA; 3 Chungnam National University, Daejeon, Korea

**Keywords:** Animal Monitoring, Decision Support Systems, Hybrid Intelligent Mechanistic Models, Precision Nutrition, Smart Livestock Farming, Sustainable Livestock Management

## Abstract

Precision Livestock Farming (PLF) has evolved dramatically from basic monitoring systems to sophisticated artificial intelligence (AI)-driven decision support systems that enhance livestock management efficiency, sustainability, and animal welfare. This review examines the technological evolution of PLF since 2017, highlighting significant advancements in sensing technologies, computer vision, and AI. Non-invasive technologies, including red-green-blue and depth cameras, 3D imaging systems, and Internet of Things-enabled platforms, now capture detailed biometric and behavioral data in real time, while AI algorithms enable early disease detection, optimize feeding strategies, and improve reproductive management. Integrating these technologies with mechanistic models has created hybrid intelligent frameworks that address longstanding challenges in precision nutrition modeling. Future PLF development will likely focus on integrating large language models, adopting federated learning approaches to address data privacy concerns, and democratizing technologies for small-scale producers. Despite technological progress, challenges remain regarding data standardization, connectivity in rural environments, high implementation costs, and ethical considerations around increased animal monitoring. By fostering interdisciplinary collaboration among animal scientists, engineers, computer scientists, and social scientists, PLF can continue to drive sustainable and efficient practices in livestock production while ensuring that technologies complement rather than replace traditional husbandry knowledge.

## INTRODUCTION

The concept of Precision Livestock Farming (PLF) emerged as a framework to enhance livestock management through the use of advanced monitoring technologies, with early contributions from researchers such as Daniel Berckmans [1]. PLF emphasizes a “per animal” approach, where sensor technologies, artificial intelligence (AI), and big data analytics enable real-time monitoring and precise management of individual animals. This data-driven approach optimizes health, welfare, and productivity, moving beyond traditional herd-level management to tailor decision-making to each animal’s unique needs [2]. This foundational work laid the groundwork for integrating PLF into broader frameworks such as Smart Livestock Farming (SLF), which expands PLF’s capabilities by incorporating Internet of Things (IoT)-enabled platforms, cloud computing, and machine learning (ML)-based decision-support systems (DSS). While often discussed together in modern agricultural literature, PLF and SLF are distinct yet complementary approaches that share common goals, with SLF representing an evolution that incorporates PLF methodologies within a broader and more automated technological ecosystem. Together, PLF and SLF are designed to enhance individual animal management while addressing pressing industry challenges such as environmental sustainability, animal welfare, and resource-use efficiency [3,4].

The evolution of PLF has been extensively documented, with Berckmans’ book Advances in PLF [5] providing a comprehensive review of recent progress. The transformative potential of PLF technologies spans across many fields, enabling the creation of climate-smart livestock systems that address critical issues such as disease prevention, improved feed efficiency, and reduced environmental impact. Key advancements in on-animal sensors, thermal imaging, machine vision, and acoustic monitoring have enabled early detection of health concerns such as mastitis, lameness, fertility disorders, and metabolic diseases [6]. Additionally, automated feeding systems, real-time grazing management, and AI-driven milking technologies have enhanced livestock productivity and welfare, making modern PLF more scalable and accessible [7]. In particular, rumen biosensors, which use potentiometric and ion-sensitive field-effect transistor (ISFET) technologies, have allowed automated continuous monitoring of ruminal pH, temperature, and possibly volatile fatty acid concentrations, enabling precise metabolic tracking [8,9].

Since these foundational contributions, technological advancements have continued to reshape PLF. A significant shift has changed not only since the initial conceptualizations but also since our literature review in 2017 [10]. The rise of AI, particularly large language models (LLM), has made complex data interpretation more accessible, transforming PLF from a passive monitoring tool into an intelligent, predictive DSS. AI-powered convolutional neural networks (CNN) and recurrent neural networks (RNN) are now capable of detecting subtle deviations in animal behavior, feeding patterns, and movement through motion-tracking techniques (i.e., video), enabling early disease detection before clinical symptoms appear [11,12]. Similarly, sensor technologies have become more precise, affordable, and diverse, enabling broader adoption across different scales of livestock production. Integrating these technologies has elevated PLF and SLF into critical components of modern livestock systems, enhancing productivity while addressing animal welfare and environmental sustainability.

While PLF and SLF represent significant advancements in livestock management, their adoption does not inherently guarantee sustainability across environmental, economic, or social dimensions. Sustainability is a multifaceted concept requiring careful consideration of the complex interactions within production systems [13]. Implementing PLF may lead to environmental benefits, such as improved resource efficiency and reduced methane emissions, but it also entails significant economic costs associated with technology adoption and maintenance. Moreover, integrating such advanced technologies could disrupt social sustainability, potentially marginalizing smallholder farmers or those unable to afford these innovations. Thus, while PLF offers a promising pathway toward sustainability, it must be coupled with strategies to ensure its benefits are equitably distributed, its costs are manageable, and its adoption does not inadvertently exacerbate existing disparities in the livestock sector. These considerations underline the importance of a systems approach in evaluating the broader implications of PLF within diverse production contexts.

This review builds on our earlier work and incorporates insights from recent developments in PLF, particularly in sensing technologies, computer vision (CV), and AI. We also discuss the evolution of PLF technology, ethical considerations, and limitations, provide context for linking these advancements with practical applications, and outline future research directions to address ongoing challenges in livestock production systems.

## THE EVOLUTION OF PRECISION LIVESTOCK FARMING TECHNOLOGIES

**Figure 1:**
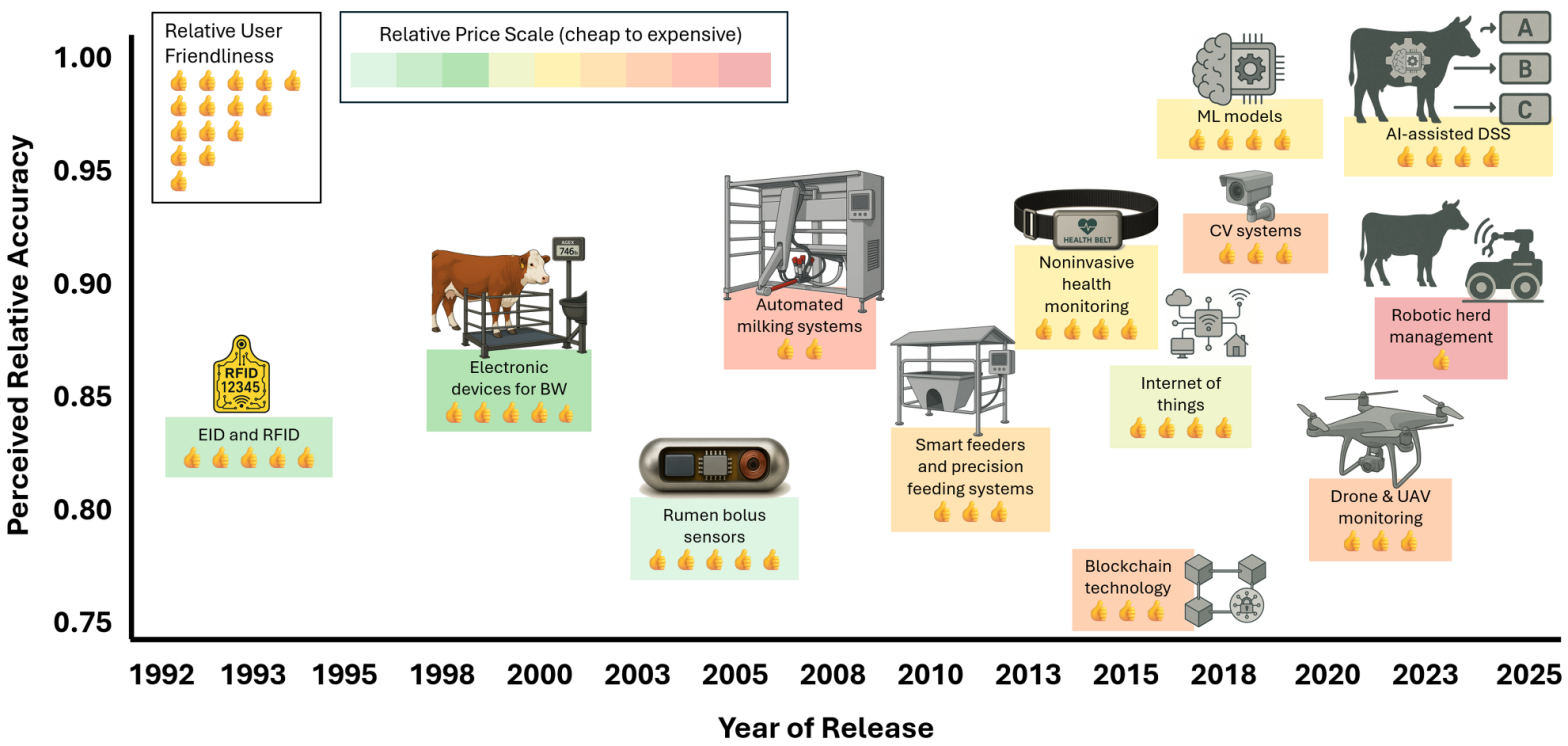
Evolution of precision livestock farming technologies (1992–2025) regarding their perceived relative accuracy, user-friendliness, and cost towards improving precision animal nutrition. EID, electronic identification; RFID, radio-frequency identification; BW, body weight; ML, machine learning; CV, computer vision; UAV, unpersoned aerial vehicle; AI, artificial intelligence; DSS, decision support systems.

The PLF has evolved from basic monitoring systems to sophisticated AI-driven DSS, enhancing livestock management efficiency, sustainability, and animal welfare. Figure 1 presents a comprehensive evolution of PLF technologies representing a remarkable journey spanning three decades, with significant transformations in capability, accessibility, and implementation. A comprehensive visualization of this evolution reveals the interrelationship between three critical dimensions—accuracy, user-friendliness, and cost—providing valuable insights into adoption patterns and technological progression.

### Historical development and technical evolution

Initially, PLF relied on electronic identification (EID), wearable sensors, and automated milking systems to track animal health and productivity [2]. These foundational technologies from the early 1990s established the first generation of precision management tools, with radio-frequency identification (RFID) systems showing moderate accuracy (approximately 0.84) but high user-friendliness ratings, as indicated by their widespread adoption. The mid-2000s marked a significant advancement with the introduction of rumen bolus sensors and automated milking systems, which improved data collection capabilities but required substantial investment. By the 2010s, the rise of AI, IoT-based remote monitoring, and CV-enabled real-time detection of feeding behaviors, lameness, and disease outbreaks [14]. This period also saw the emergence of blockchain technology and advanced IoT applications, expanding the scope of PLF beyond individual animal monitoring to encompass entire production systems. In 2017, PLF systems primarily relied on wearable sensors to measure parameters such as body weight (BW), milk yield, and behavioral metrics like feeding and rumination patterns. These systems laid the groundwork for precision management but were often constrained by the need for manual data collection and interpretation [15,16]. The graphical sketch in Figure 1 confirms this assessment, showing wearable technologies with improved accuracy (0.85–0.90) compared to earlier systems but with varying degrees of user-friendliness. While not directly comparable to earlier systems, such as RFID weight scales, which serve different functions, wearable systems still reflect an overall trend toward improved precision in sensor technologies, albeit with varying degrees of user-friendliness.

### Recent advancements and the current state

Over the past few years, non-invasive technologies have emerged as the new standard. Red-green-blue and depth (RGB-D) cameras and 3D imaging systems now capture detailed biometric and behavioral data in real-time, eliminating many of the limitations of earlier tools [6]. As depicted in Figure 1, CV systems show perceived high accuracy ratings (0.93–0.94) with moderate user-friendliness (i.e., 3 thumbs-up), representing a technological leap while maintaining accessibility. Advancements in rumen biosensors, such as potentiometric and ISFET-based pH sensors [17], have significantly improved the precision of monitoring key metabolic parameters like pH and temperature [18]. Han et al [8] discuss how these sensors leverage low-power, wide-range wireless communication technologies, such as low-power, long-range wide area network (LoRaWAN), to enable real-time data transmission, paving the way for more accessible and scalable PLF applications. Integrating IoT devices with cloud-based analytics platforms has revolutionized data acquisition, making real-time decision-making possible. This advancement is supported by AI-powered ML or deep learning algorithms (e.g., CNN, RNN, YOLO-based) that can now forecast disease outbreaks and detect subclinical health issues before they manifest [19,20]. Our visualization shown in Figure 1 assigns these technologies the highest accuracy ratings (0.95–0.99) in the PLF domain [21–23], though with varying levels of user-friendliness and implementation cost [24,25]. While CV systems exhibit high visual detection accuracy, integrated ML-DSS leverage multimodal data, including CV, biosensor, and environmental input, resulting in superior accuracy (0.95–0.99) for complex predictive tasks such as early disease forecasting. When evaluating ML and AI-assisted DSS systems in livestock management, several critical considerations must be carefully weighed beyond the impressive accuracy metrics displayed in the visualization. These systems often demonstrate high performance within controlled evaluation environments using carefully curated datasets that may not fully represent real-world farm operations’ complex, dynamic conditions. The black-box nature of many sophisticated ML models creates a fundamental tension between accuracy and explainability, potentially undermining farmer trust despite technical excellence. Additionally, these systems exhibit significant data dependency, where performance can degrade substantially when deployed in environments with characteristics different from those of their training data. This highlights the importance of comprehensive validation across diverse operational contexts and seasons before drawing definitive conclusions about their superiority over other technologies like CV systems, which may offer better robustness despite slightly lower benchmark accuracy scores.

### Adoption considerations and barriers

Despite technological advancement, user-friendliness has not necessarily improved at the same rate as accuracy, with some newer technologies presenting significant learning curves for farmers. Research by Silvi et al [26], who surveyed 378 Brazilian dairy farms, found that farmers rated “user-friendliness” as the third most important factor (4.39 out of 5) in technology adoption decisions, following only “available technical support” (4.55) and “return on investment” (4.48). The visualization’s color-coding for cost (green for affordable, yellow for moderate, and red for expensive) in Figure 1 reveals that the most advanced technologies generally entail higher implementation costs. Automated milking systems, blockchain applications, drone monitoring, and robotic herd management fall into the expensive category, potentially limiting their adoption despite their superior accuracy. Silvi et al [26] confirmed this cost barrier, finding that the most frequent reason farmers cited for not investing in precision technologies was “the need for investment in other sectors of the farm” (36%), followed by “uncertainty of return-on-investment” (24%).

Practical implementations of PLF and SLF demonstrate how these technologies can contribute to sustainability across environmental, economic, and operational domains. Environmentally, PLF has been shown to enhance resource efficiency, optimize feeding strategies, and lower methane emissions through improved monitoring and data-driven decision-making [18]. In the feedlot, PLF can minimize environmental impact while increasing profitability through real-time monitoring of livestock health, behavior, and performance while supporting greenhouse gas reduction, resource optimization, and improved animal welfare by implementing advanced feed management, environmental monitoring, individualized feeding protocols, early disease detection, and waste management solutions—ultimately supporting more sustainable feedyard operations [27]. In addition, satellite-integrated grazing systems, combined with on-ground IoT sensors, enable adaptive grazing strategies, reducing overgrazing and soil degradation [28–32]. However, according to the economic viability, technology adoption and maintenance costs pose challenges, particularly for smallholder farmers or operations with limited financial resources [8]. Public-private partnerships and government incentives have been proposed to lower economic barriers to PLF adoption [33]. Therefore, one of the main points of improvement is the affordability of new technologies by small-scale farmers and ranchers.

Regarding ethical and social implications, research suggests that balancing real-time monitoring with animal well-being is crucial. Automated behavior analysis, using AI-driven metrics such as heart rate variability, lying time, and feeding patterns, could ensure that PLF systems align with welfare standards rather than disrupt natural behaviors [3]. The integration of AI-driven monitoring raises ethical concerns regarding constant surveillance. Continuous tracking of physiological and behavioral parameters could potentially induce stress in animals if not implemented with animal welfare considerations in mind; therefore, all the new emergent technologies should consider this point, maybe replacing wearable sensors (such as big collars) with other non-wearable or non-invasive devices, such as cameras [34]. Also, some argue that PLF adoption may weaken the human-animal bond, negatively affecting animal welfare [35]. As farmers increasingly rely on sensors and robots, opportunities to observe, touch, and emotionally connect with animals inevitably decrease. This may lead to a tendency to regard animals merely as commodities for food production. A lack of regular human interaction can make human presence a source of animal stress. Conversely, for farmers, reduced contact with animals limits their ability to understand animal physiology and behavior better. Moreover, since PLF systems heavily depend on electronic devices, power outages or equipment malfunctions could result in prolonged distress or harm to the animals.

## APPLICATIONS OF PRECISION LIVESTOCK FARMING IN LIVESTOCK SYSTEMS

### Predicting feed intake and feeding behavior

Feed intake is a critical variable for livestock management, influencing both productivity and environmental impact. Traditional methods of measuring dry matter intake (DMI) often relied on manually weighing feed, which was labor-intensive, prone to human error, and time-consuming. Recent advancements in CV systems have transformed this process. RGB-D cameras can now analyze feed mass and volume changes, while AI models interpret behaviors such as chewing and biting patterns to estimate individual feed intake with remarkable accuracy [11]. For example, studies using CNN have reported mean absolute errors as low as 0.1 kg for DMI predictions [36]. Most of the CV algorithms used to predict DMI analyze feed disappearance rates by tracking changes in feed volume and mass; however, new real-time algorithms can track the individual feeding duration, bite rate, and rumination patterns [37] for personalized feeding programs tailored to individual animal needs. In addition, these AI-powered early warning systems alert producers to abnormal feeding behaviors, allowing rapid intervention in cases of illness or stress [38].

The main issue with these algorithms is that they often rely on estimated ingestion time, which can be misrepresented. For instance, ingestion time is commonly recorded as the interval between the animal’s arrival and departure from the feeder, although animals may remain at the feeder without actively ingesting. While recent ML techniques aim to correct this by detecting feeding activity based on weight changes in the feeder, the accuracy of feed intake prediction still varies. Davison et al [39] showed that despite corrections for BW and dietary characteristics (e.g., moisture, energy, and fiber), prediction performance remains modest for practical application. However, a consensus is emerging that incorporating dietary parameters into the models significantly enhances prediction accuracy [40]. Feeding behavior traits—such as ingestion time, bite rate, and jaw movement—also improve model performance. For instance, Ding et al [41] estimated individual feed intake based on jaw movement using a triaxial accelerometer and reported high accuracy (R^2^ = 0.97 with root mean square error [RMSE] = 0.36). However, it is important to note that many reported high-accuracy values are obtained under controlled experimental conditions or with limited datasets, and often fail to generalize to diverse commercial environments. As emphasized in some studies [42,43], the accuracy of ML models depends heavily on the similarity between training and testing data, underscoring the need for independent validation across broader production systems.

### Estimating body weight and body condition score

Accurate estimation of BW and body condition score (BCS) is essential for evaluating animal health, productivity, and welfare. Modern CV-based PLF solutions have made it possible to non-invasively estimate BW and BCS with remarkable accuracy [44]. Correlation coefficients exceeding 0.9 have been reported in several studies [6]. These technologies allow producers to monitor BW and BCS non-invasively, reducing animal stress and labor demands. This level of accuracy has been possible thanks to the new advanced RGB-D cameras and ML models extracting body contour data to accurately estimate BCS without human intervention. In addition, IoT-connected to AI models enable accurate real-time weight monitoring in cattle [45]. Determining both the BW and the BCS of animals was one of the first objectives of the new ML algorithms [46]; therefore, the performance acquired is already high, but there is room for improvement. In this sense, the next step is to predict individual BW of non-confined animals. Some drone-based or unpersonned aerial vehicles (UAV)-assisted works have already been developed to estimate the BW of cattle in the pasture [47–49]. This shows the vast emerging potential of CV algorithms coupled with drones and other devices allowing them to track animals on grazing conditions.

### Management of reproduction

Precision technologies have been used in reproduction for livestock production to increase fertility by precise estrus detection, execution of artificial insemination, assessment of BCS before breeding, regulation of the voluntary waiting period, and monitoring of calving rates along with associated complications [50–52]. Among all these actions where precision technologies have been used, efficient estrus detection is a critical component of reproductive management in dairy and beef cattle systems. It represents the first point before all posterior reproductive treatments, such as synchronization or artificial insemination [53]. The ability to accurately identify cows in estrus directly influences conception rates, calving intervals, and overall reproductive efficiency. Traditional methods, such as visual observation of estrus signs (e.g., standing heat, increased activity, and vocalization), are labor-intensive and have limited accuracy due to the increasing prevalence of silent heat in high-yielding dairy cows [54]. As a result, PLF technologies have emerged as reliable solutions for automating estrus detection through continuous and objective monitoring of animal behavior, physiological parameters, and hormonal changes.

One of the most widely used technologies for estrus detection involves wearable sensors that measure cow movement and activity levels. Studies have demonstrated that cows in estrus exhibit a 2- to 4-fold increase in movement, including restlessness, walking, and mounting behavior [55]. Devices such as pedometers, accelerometers, and gyroscopes integrated into collars, ear tags, or leg-mounted sensors detect these behavioral changes. Several studies demonstrated how collars [56] and accelerometers [57] can be used to determine estrus and link it with some nutritional aspects. Estrus affects physical activity and alters feeding and rumination patterns due to increased restlessness. Studies show that cows reduce their rumination time by 10% to 30% before estrus onset, which can be detected using bolus sensors, jaw movement monitors, or integrated smart collars [58].

Systems such as SCR tag (cSense Flex tag, SCR Engineers) and CowManager (Agis Automatisering) analyze changes in feeding behavior, improving estrus detection when combined with activity data. ReithandHoy [57] found that integrating rumination data with activity sensors increased estrus detection accuracy from 85% to 95%, while Pahl et al [58] showed that a 30% drop in rumination was a strong predictor of estrus when used in combination with motion-based estrus detection. Brehme et al [59] showed how temperature fluctuations 24 h pre-estrus measured through vaginal and intra-ruminal temperature sensors were reliable predictors of ovulation, with an accuracy exceeding 85%. Recent advances in CV and ML are being integrated into estrus detection systems. AI-powered video analysis detects mounting behavior, tail-raising, and vulvar swelling, improving estrus detection in large-scale operations, but can also detect changes in vocalization frequency during estrus when associated with ML-based sound analysis [60,61].

### Health monitoring and early disease detection

Health monitoring has also seen significant advancements, mainly through integrating AI with thermal and RGB-D imaging systems. For instance, thermal imaging combined with YOLO algorithms has achieved over 87% accuracy in detecting mastitis [62]. Mastitis is one of the most common and costly diseases in dairy cattle, and early detection through thermal imaging helps reduce antibiotic usage, improve milk quality, and enhance overall herd welfare. Multiple studies have validated this method, such as Sejian et al [63], who demonstrated how ML algorithms trained on infrared data can distinguish infected udders with higher precision than manual inspections. Furthermore, AI-based imaging can detect other inflammatory conditions, such as foot rot and lameness, providing a broader scope of disease prevention through non-invasive temperature monitoring [64]. Regarding temperature analysis, other studies utilized deep learning and neural networks to prevent heat stress in livestock [65,66].

Another key development in AI-powered disease monitoring is the detection of bovine respiratory disease (BRD), a leading cause of morbidity and mortality in beef cattle. AI-based thermal analysis of nasal temperatures and breathing patterns has allowed researchers to identify abnormal respiratory rates that signal early-stage BRD, often days before clinical symptoms appear [19]. A study by Ghassemi Nejad et al [67] confirmed that thermal imaging cameras placed near feeding areas can continuously track nasal heat fluctuations, providing early warning alerts to farm managers. The ability to diagnose subclinical respiratory issues before visible distress develops significantly improves treatment outcomes, reduces disease transmission, and lowers economic losses due to BRD-related fatalities [68]. These tools are transforming animal health management by shifting the focus from reactive to proactive care, minimizing the excessive use of antibiotics.

Beyond external imaging, wearable and ingestible sensor technologies have transformed internal health monitoring in cattle. Rumen sensors, particularly those designed for continuous monitoring of ruminal pH and motility, have played a crucial role in the early detection of digestive disorders, especially since incorporating new ML techniques to analyze the output data [69]. The rumen is the central organ in cattle fermentative processes, and imbalances in pH levels can indicate conditions such as subacute ruminal acidosis (SARA), bloat, and metabolic inefficiencies. Several studies [18,70] demonstrated that wireless telemetry systems tracking pH fluctuations provide real-time insights into rumen function, helping farmers optimize feeding strategies and prevent metabolic diseases. These systems are particularly effective in precision feeding, where AI models adjust dietary compositions based on real-time pH fluctuations, ensuring optimal microbial fermentation and nutrient absorption. Phillips et al [71] demonstrated the effectiveness of wireless telemetry systems in tracking ruminal pH, highlighting their potential in monitoring SARA over extended periods.

Nonetheless, ruminal pH boluses face several critical performance limitations that might impair their reliability as diagnostic tools in ruminant nutrition. Electrochemical pH sensors are particularly susceptible to biofouling from protein-rich diets, as proteins readily adsorb to sensor surfaces through hydrophobic interactions and electronic attraction, creating interference layers that compromise signal detection and accuracy [72]. This protein-induced biofouling might be especially problematic in ruminants fed high-concentrate or high-protein diets. Additionally, substantial sensor drift over time presents another significant challenge, with observed drift patterns requiring frequent recalibration to maintain measurement accuracy [18,73]. Despite manufacturer claims of extended operational lifespans, research consistently demonstrates that these devices’ practical pH monitoring capabilities typically deteriorate after approximately 80–90 days of deployment, significantly shorter than the often advertised 150+ days of functionality [8]. Despite these limitations, ruminal pH monitoring systems have shown transformative potential for continuous rumen surveillance. Recent advancements in wireless telemetry and sensor design, such as low-power electronics, improved coating materials, and more robust signal calibration, could help mitigate issues like sensor drift and biofouling, while extending battery life and enhancing data transmission. As such, these technologies remain a promising tool for commercial livestock systems, provided that future iterations continue to address the practical challenges associated with long-term deployment under diverse dietary and environmental conditions.

Our research has extensively used rumen sensors to analyze the area and time above and under the curve to detect abnormal ruminal pH patterns that might lead to SARA [74–78]. The area and time under the pH-time curve [130] and redox potential can provide key insights into fermentation dynamics and microbial activity. Such metrics aid in the early detection of SARA and enable precise adjustments in dietary formulations to optimize microbial efficiency, supporting sustainable livestock systems. However, one major challenge in widely adopting ruminal pH sensors has been long-term calibration accuracy and sensor costs. As mentioned above, Neubauer et al [73] highlighted that indwelling pH sensors suffer from signal drift, requiring frequent recalibration to maintain precision. To address this issue, recent advancements (not related to livestock science) in self-calibrating sensor technologies have introduced AI-driven automatic recalibration mechanisms, significantly improving data reliability for long-term applications [79].

While these AI-enabled adjustments improve technical performance, they also introduce a new layer of complexity: the raw pH data are often processed through proprietary algorithms managed by third-party service providers. As a result, the end-user typically receives only interpreted outputs rather than access to the raw sensor readings or the details of the data transformation pipeline [80]. This “black box” scenario limits transparency and prevents independent validation of physiologically meaningful patterns, raising concerns about data ownership, reliability, and the long-term sustainability of such systems. If commercial providers discontinue services or restrict access through licensing models, the practical application of these technologies in both research and production settings could be compromised [80].

Additionally, Han et al [8] emphasized the scalability of LoRaWAN technology, which facilitates seamless data transmission in both large-scale and smallholder farms. This innovation significantly enhances the accessibility of advanced sensor systems. Han et al [8] also highlighted the importance of integrating rumen motility and temperature sensors with pH sensors. These combined datasets improve diagnostic accuracy and provide actionable insights for managing critical conditions such as ruminal tympany, displaced abomasum, and mastitis. By leveraging multiple sensor parameters, these integrated systems empower producers to make timely, data-driven decisions, improving both animal welfare and production efficiency, as long as battery life is improved. The advancements in AI-driven cattle health monitoring extend beyond disease detection, influencing overall herd management strategies. For instance, AI-based estrus detection systems have enhanced reproductive efficiency by accurately identifying heat cycles in cows through motion tracking, temperature variations, and behavioral analysis [40]. These automated monitoring tools eliminate the need for manual estrus detection, reducing missed breeding opportunities and optimizing artificial insemination schedules. Similarly, CV algorithms combined with wearable accelerometers are being used to detect early signs of lameness and locomotion disorders, ensuring timely intervention and reducing culling rates in dairy operations [81].

Finally, as AI-powered biosensors, imaging tools, and deep learning models continue to evolve, the future of precision livestock health management is shifting toward fully autonomous, predictive healthcare systems. AI-driven platforms are increasingly capable of analyzing multi-source sensor data, forecasting disease risks, and recommending real-time preventative actions. Research by Fuentes-Peñailillo et al [82] suggests that integrating blockchain-based AI systems could further enhance data security and traceability in livestock health monitoring, ensuring greater transparency in disease management and food safety regulations. The widespread adoption of AI-driven health monitoring systems transforms modern cattle farming by providing earlier disease detection, reducing reliance on antibiotics, enhancing overall herd productivity, and making the veterinary profession in livestock easier [83]. These innovations ensure a shift toward sustainable, efficient, and welfare-conscious livestock production, paving the way for precision agriculture and data-driven animal health management.

## PRECISION NUTRITION MODELING IN LIVESTOCK SYSTEMS

Precision nutrition modeling represents a transformative approach to livestock management, leveraging advanced technologies and computational methods to optimize animal nutrition at the individual level. This section explores the evolution of nutrition modeling from population-based approaches to personalized feeding strategies, examining the integration of sensors with mechanistic models, hybrid modeling approaches, data-driven methodologies, DSS, and future directions in this rapidly evolving field.

### Integration of mechanistic models with precision livestock farming technologies

The incorporation of AI into PLF represents a paradigm shift in livestock management. Neural networks, particularly CNN and RNN, excel at extracting meaningful patterns from complex datasets, making them invaluable for image classification, object detection, and segmentation tasks. Some CV algorithms have proven particularly effective in automating labor-intensive processes, from tracking feeding behavior to identifying early signs of disease [6]. Table 1 summarizes some of the most relevant livestock-related work in AI. These AI capabilities have particular relevance for precision nutrition modeling, where individual animal feeding behavior and metabolic responses must be accurately monitored and predicted.

Recent advances in miniaturized, low-power sensors have dramatically expanded the physiological parameters that can be monitored in real-time. Modern sensing technologies deployed in livestock systems can monitor various parameters, including feeding behavior, physiological status, and movement patterns, through IoT devices that collect real-time data [84,85]. When coupled with advanced nutrition models, these sensors allow for real-time dietary adjustments that optimize rumen function on an individual animal basis. Such continuous monitoring capabilities represent a significant advance over traditional methods that rely on periodic sampling and population averages.

Traditional nutrition models, such as those published by the National Academies of Sciences, Engineering, and Medicine (NASEM), provide valuable frameworks but often rely on population averages rather than individual animal characteristics. Integrating real-time data from PLF technologies with mechanistic nutrition modeling [86–89] represents a significant paradigm shift, moving from generalized recommendations toward individualized, dynamic nutritional management that fuses data with pre-established concepts and visceral understandings of scientific knowledge.

**Figure 2:**
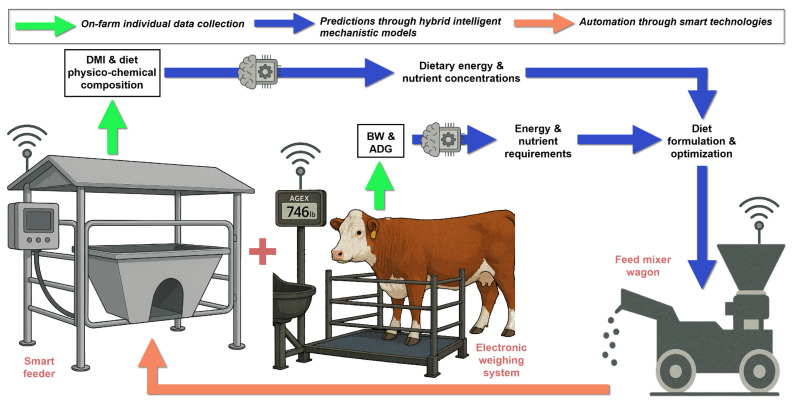
Diagram showing the integration of precision livestock farming technologies with artificial intelligence and mechanistic modeling to increase feed efficiency through precision nutrition techniques. DMI, dry matter intake; BW, body weight; ADG, average daily gain.

The advancement of AI has transformed nutrition modeling from passive monitoring into intelligent, predictive DSS. Neural networks can detect subtle deviations in animal behavior, feeding patterns, and movement, enabling early detection of metabolic changes before clinical symptoms appear. Alonso et al [84] developed an intelligent edge-IoT platform for monitoring livestock and crops in a dairy farming scenario that demonstrated how these technologies could be integrated to provide real-time decision support. This real-time monitoring capability has profound implications for precision nutrition models, allowing for dynamic adjustment of feeding strategies based on individual animal responses rather than population averages. For instance, Pomar et al [90] developed a system where some “smart” technologies measured the DMI of growing pigs, calculating daily energy and protein requirements and predicting these requirements for the following days (i.e., forecasting). After this, a smart feeder distributes the ration according to individual needs daily, increasing feed efficiency by reducing feeding costs and decreasing nitrogen excretion [91]. Through new PLF technologies, such as the electronic weight scale and the smart feeders, it is possible to estimate animal requirements and, posteriorly, considering observed intake, estimate the energy and protein concentration required in the diet (Figure 2). Like our concept shown in Figure 2, Awasthi et al [92] developed an ML simulation model to predict average daily gain (ADG) in pasture-based beef cattle using autonomously collected walk-over weights. An XGBoost model was trained on cleaned data incorporating age, sex, breed, and weather conditions. The model successfully simulated ADG, showing strong agreement with observed values. The mean difference between simulated and measured BW was −1.2 kg with a standard deviation of 27.3 kg. The ADG patterns were realistically reproduced, supporting the model’s utility for herd growth monitoring and management decision-making. In summary, PLF technologies will be a key part of providing individual on-farm data from animals, and this data will feed the database, which through mechanistic models will provide information (e.g., predictions, requirements, early warnings), helping the decision-making process in animal production of cattle farmers and improve their efficiency and sustainability.

### Hybrid intelligent mechanistic models for nutrition

Developing hybrid intelligent mechanistic models (HIMM, [93,97]) represents the cutting edge of precision nutrition modeling. Combining AI with mechanistic models has opened new frontiers in predictive analytics. While mechanistic models excel at simulating livestock responses under controlled conditions, they often lack the flexibility to account for real-world variability. Hybrid models that integrate data-driven insights from AI with mechanistic frameworks bridge this gap, providing robust tools for optimizing livestock performance and resource use. These hybrid approaches can address longstanding challenges in nutrition modeling, such as the difficult-to-measure parameters that have traditionally limited model accuracy. Thus, combining AI with mathematical models has led to hybrid frameworks capable of predicting livestock responses to dietary changes, optimizing feed efficiency while reducing nutrient waste [93]. For example, passage rate has been identified by Allen [94] as perhaps the most significant limiting factor in predicting nutrient digestibility in the rumen. HIMM can address this challenge through two approaches: (1) embedding AI within mechanistic models to predict variables such as passage rates that are affected by multiple factors, or (2) embedding mechanistic models within neural networks to strengthen AI predictions with bio-physicochemical foundations.

The concept of “digital twins” for dairy cows has emerged as an innovative application of hybrid modeling approaches. Neethirajan and Kemp [95] describe digital twins as digital replicas of real-world entities that simulate physical and biological states based on input data, helping with prediction, optimization, and decision-making. These virtual representations combine sensor data with mechanistic nutrition models to simulate different dietary scenarios before implementation, allowing for personalized feeding strategies that account for individual variations in metabolism, production stage, and health status. This approach demonstrates how hybrid models can bridge the gap between theoretical nutrition science and practical farm implementation.

Validation of HIMM presents unique challenges due to their hybrid nature. Traditional statistical validation metrics may not fully capture model performance across the conditions encountered in commercial livestock operations. Furthermore, with the increasing availability of big data, the assumptions underpinning classical statistical inference may no longer be valid [131]. In large datasets, even trivial effects can become statistically significant, potentially leading to overfitting or spurious conclusions, thereby necessitating new validation paradigms tailored to complex, high-dimensional data. Research in digital twin implementations for livestock suggests that multi-level validation frameworks are needed to assess both predictive accuracy and biological plausibility through independent datasets and expert evaluation [96]. Such approaches emphasize the importance of validating output predictions and intermediate mechanistic variables to ensure the model correctly represents the underlying biological processes.

### Data-driven approaches to enhance mechanistic nutrition models

The emergence of data analytics has evolved through multiple stages, from basic descriptive analytics to predictive and prescriptive analytics and ultimately to *smart learning* systems. These increasingly sophisticated approaches move from simply collecting and responding to data to predicting and prescribing actions and finally to smart learning and policy making [97].

In nutrition modeling, data-driven approaches have been particularly valuable for addressing variables that are difficult to measure directly. For example, research on predicting DMI has shown that adding dietary parameters to ML models significantly increases prediction accuracy. Similarly, Ding et al [41] demonstrated high accuracy (R^2^ = 0.97) in estimating individual feed intake based on jaw movement measured through triaxial accelerometers. However, as discussed above, independent validation using external datasets is necessary to confirm whether such high accuracy can be consistently achieved across diverse production systems.

The integration of non-traditional data sources has expanded the predictive capabilities of nutrition models. Recent research by Monteiro et al [98] demonstrates how AI approaches with feature engineering and ensemble methods can utilize rumen microbiome data to predict feed efficiency in dairy cows. Their study showed that the rumen microbiome plays a pivotal role in explaining variance in milk fat and protein production efficiency, potentially reducing methane emissions by up to 37.5% through selection for better residual feed intake. Similarly, voice analysis of livestock vocalizations shows promise as an early indicator of metabolic stress, offering a novel data stream for precision nutrition systems.

Multi-omics approaches represent another frontier in data-driven nutrition modeling. Fontanesi [99] highlights that metabolomics provides valuable insights for livestock genomics and phenotyping applications, allowing for identifying biomarkers related to productive traits. Novais et al [100] applied factor analysis and Bayesian network modeling to integrate different omics data for studying production, carcass, and meat quality traits in cattle, demonstrating how multi-level data integration can reveal non-obvious relationships that exist among omics data. This multi-omics integration allows for identifying biomarkers of metabolic efficiency that can be incorporated into predictive models.

These data-driven approaches do not replace traditional mechanistic models but rather enhance them by improving parameter estimation and expanding their predictive capabilities. As Tedeschi [97] noted, “Success and failures in model building are more related to the ability of the researcher to interpret the data and understand the underlying principles and mechanisms to formulate the correct relationship among variables rather than profound mathematical knowledge.”

### Decision-support systems for precision nutrition management

The ultimate goal of precision nutrition modeling is to develop DSS that transform complex data into actionable feeding recommendations. As Lee et al [101] demonstrated in their groundbreaking work with ML models for metabolizable protein supply prediction, combining traditional mechanistic nutrition principles with advanced AI techniques can dramatically improve prediction accuracy. Their research showed that support vector regression and random forest models significantly outperformed conventional NASEM [87] equations, with R^2^ values of 0.76 for microbial nitrogen and 0.60 for rumen-undegradable protein compared to just 0.04 and 0.27, respectively, for traditional methods.

These DSS must balance scientific rigor with practical implementation, providing clear guidance to livestock managers while accommodating operational constraints. Modern DSS integrate multiple data sources, including feed composition analysis, individual animal monitoring (via sensors and CV), environmental conditions, and economic parameters. For ruminants, DSS must account for the complex interactions between dietary composition, ruminal fermentation, and metabolic processes. Integrating real-time rumen monitoring data into nutrition models allows DSS to dynamically adjust feeding recommendations based on actual fermentation patterns rather than assumptions. This creates a feedback loop where dietary adjustments can be fine-tuned based on individual animal responses, moving beyond the population-average approach of traditional nutrition models.

User interface considerations are critical for DSS adoption in commercial settings. Research by Eastwood et al [102] emphasizes the importance of farmer-centric design approaches for precision dairy technologies, highlighting that systems must align with farmers’ existing practices and decision-making processes. Similarly, Groenendaal et al [103] demonstrated that visualization approaches that highlight economically significant deviations from expected outcomes, rather than presenting raw data, significantly improved user engagement with nutrition decision support tools. Wolfert et al [104] further emphasize that successful smart farming applications must translate sophisticated data outputs into actionable insights that farm managers can readily implement without specialized data science training. This human-centered design approach represents an important advance in translating complex modeling outputs into practical on-farm actions.

The economic value proposition of precision nutrition DSS extends beyond feed cost savings. According to Zuidhof [105], precision livestock feeding aims to match nutrient supply precisely with the nutrient requirements of individual animals based on real-time feedback from sensors, providing benefits including greater economic returns, reduced environmental excretion, and improved resource utilization efficiency (Figure 2). Multiple studies have demonstrated improved income over feed costs, with additional benefits in reduced veterinary expenses due to improved metabolic health. Importantly, farms have shown a positive return on investment within months of implementation, with smaller operations experiencing longer payback periods but similar percentage improvements in profitability.

The development of effective DSS represents a crucial intersection where sensor technology meets nutrition modeling. Building on the advances reported by Lee et al [101], incorporating physiological parameters and real-time sensor data within HIMM illustrates the power of combining biological insights with AI capabilities. Such integration enables models to retain mechanistic interpretability while achieving higher predictive accuracy. These systems benefit from the increasingly large volumes of data collected through PLF technologies, transforming them into actionable insights. HIMM offer a promising approach to managing individual animal variation without sacrificing scientific rigor, supporting adaptive nutrition management that dynamically adjusts to changes in metabolic status and environmental conditions. These systems have shown the potential to reduce feed costs by 7% to 12% and decrease nutrient waste, aligning with both economic and sustainability objectives.

By integrating sensor data with mechanistic models and AI algorithms, precision nutrition DSS can enable adaptive feeding strategies that account for individual animal requirements, metabolic status, and environmental conditions. Such systems optimize resource use and production efficiency and contribute significantly to environmental sustainability through reduced nutrient waste and improved animal welfare.

### Future directions and challenges

While precision nutrition modeling offers transformative potential, several implementation challenges remain to be addressed. Precision nutrition faces many challenges, just as other PLF technologies do, while dealing with nutrition-specific barriers. These models must overcome technical and adoption hurdles for effective integration into commercial livestock operations. The technical challenges specific to nutrition modeling include the need for accurate, real-time data on feed composition, intake, and digestibility. Current sensors can measure intake quantity, but determining feed quality parameters in real-time remains difficult. Additionally, the biological complexity of rumen fermentation and nutrient metabolism creates significant modeling challenges that require sophisticated approaches combining mechanistic understanding with data-driven insights. Integrating nutrition models with existing farm management systems represents a significant barrier from an implementation perspective. Many producers already use various digital tools for different aspects of farm management, and nutrition models must interface seamlessly with these systems to provide true value. The development of standardized data formats and application programming interface will enable this integration and allow different technologies to communicate effectively. Economic considerations also impact adoption rates. While sophisticated nutrition modeling can improve feed efficiency and reduce waste, the initial investment in sensors, computing infrastructure, and training can be substantial. Future research must focus on demonstrating a clear return on investment pathways for different production scales and systems. This includes quantifying the economic benefits of precision nutrition beyond direct feed savings, such as improved animal health, reduced veterinary costs, and enhanced product quality.

As precision nutrition modeling advances, the ethical dimensions of data collection and use will become increasingly important. Data ownership, privacy, and security issues must be addressed through appropriate governance frameworks that protect farmer interests while enabling the collaborative data sharing necessary for model improvement. Emerging technologies like federated learning offer promising approaches to balancing these competing concerns. The ultimate success of precision nutrition modeling will depend on its seamless integration into comprehensive livestock management systems. Rather than functioning as a standalone technology, precision nutrition must become part of an integrated approach that connects feeding decisions with health monitoring, reproduction management, and environmental impact assessment. This holistic approach will enable truly sustainable livestock production systems that optimize multiple objectives simultaneously.

## LIMITATIONS, OPPORTUNITIES, AND OUTLOOK

### Limitations

While the advancements in PLF are promising and potentially transformative, several inherent challenges must be addressed to maximize their effectiveness and to ensure equitable accessibility and affordability. Key challenges include reliable connectivity for data transmission, standardization of data collection to reduce variability, questions surrounding data ownership, high implementation costs, the need for education and training of the next generation of stakeholders, and ethical concerns related to animal welfare.

One of the primary challenges lies in the real-time processing and transmission of large volumes of data generated by AI-powered monitoring systems, which demand robust computational infrastructure and reliable connectivity [80]. However, in many rural or low-resource farming environments, internet connectivity remains unstable, limiting the efficiency of cloud-based PLF solutions. One should expect that farms in remote areas often experience connectivity disruptions, leading to delays in data processing and decision-making.

One persistent issue is the variability in data quality across production environments. Factors like lighting changes, occlusions from other animals, variable sensor positioning, and camera placement can significantly impact the accuracy of CV systems [6]. In addition, changes in lighting, occlusion from other animals, and extreme weather conditions can reduce the accuracy of CV-based models [6,19,106]. Weather fluctuations (e.g., humidity, dust, extreme temperatures) add another wrinkle to consistency because they can impact the performance of sensor-based monitoring systems. During video/image processing, ML models trained on specific breeds or production systems may not generalize well across different geographical regions [27]. Another major obstacle in data accuracy is caused by sensor drift and calibration issues. For example, rumen pH sensors and temperature monitoring devices often require frequent recalibration to maintain precision in long-term data collection. Research by Nyamuryekung’e [107] showed that non-retrievable pH sensors tend to experience signal drift over time, leading to inconsistent health monitoring results. To address this, advanced self-calibrating sensors are being developed, leveraging ML algorithms to correct inaccuracies in real time. Additionally, training programs for farmers and livestock managers are essential to ensure proper calibration and maintenance protocols, thereby improving sensor reliability in practical settings.

Another key challenge that impedes the scalability of AI-driven PLF solutions is the lack of data standardization and interoperability between different sensor systems. With an increasing number of companies and research institutions developing PLF tools, data fragmentation has become a major issue. Tedeschi et al [80] emphasized that many AI-powered monitoring systems operate in isolation, using proprietary data formats that are incompatible with other platforms. This incompatibility limits cross-system integration and prevents the creation of large-scale, comprehensive datasets necessary for AI model training. To overcome this, researchers are advocating for adopting open-source PLF frameworks and standardized data exchange protocols, allowing different monitoring systems to communicate seamlessly. Related to this point, data security and privacy concerns further complicate the adoption of cloud-based AI-driven PLF technologies. Concerns over unauthorized access and data misuse have emerged, and large amounts of sensitive farm data are being collected and stored on cloud platforms. Studies by Jiang et al [108] indicate that farmers often hesitate to adopt cloud-based PLF solutions due to concerns about ownership and third-party access to livestock data. Blockchain-based data encryption technologies have been proposed as a potential solution to enhance security and transparency, ensuring that only authorized stakeholders can access farm-specific AI models.

Data ownership and security are additional issues that require attention. Adopting frameworks similar to the General Data Protection Regulation (GDPR) in agriculture could ensure that producers retain control over their data while establishing standards for secure data sharing and storage across platforms [109]. Frameworks similar to the GDPR in agriculture should be established to ensure that farmers retain control over their data while allowing for secure, standardized data sharing [108]. These regulations could prevent data misuse by third parties and ensure that farmers receive tangible benefits from the AI-driven insights generated on their farms. However, as PLF systems generate increasingly granular data, questions about who owns this information, how it can be protected from misuse, and how to store the data properly become critical issues—and farmers and ranchers might not be willing to bear this additional cost and responsibility.

Beyond infrastructure challenges, the financial burden of implementing PLF technologies remains a significant barrier, particularly for small and medium-sized farms. Some results suggest that the high initial costs of AI-driven monitoring systems, wearable sensors, and IoT-enabled devices hinder widespread adoption [110]. To alleviate this issue, public-private partnerships and government subsidies, similar to policies used in renewable energy adoption, could facilitate financial accessibility [33]. Cooperative PLF models, where multiple smallholder farms share centralized AI-driven monitoring systems, could also increase affordability and optimize resource utilization.

Beyond technological limitations, cultural and educational barriers are crucial in determining the adoption rate of AI-based PLF systems. Livestock producers lack the technical expertise to interpret AI-driven data analytics, reducing the usability of real-time health monitoring tools [111]. Addressing this gap requires comprehensive training programs, user-friendly AI dashboards, and real-time DSS that simplify complex data outputs into actionable insights. Therefore, education in mathematics and statistics and basic notions about ML and AI potentially benefit undergraduate and graduate animal science students in the livestock sector.

One of the most pressing ethical concerns is the increased livestock monitoring. While AI-driven precision tools improve disease detection, feeding management, and welfare monitoring, they may inadvertently introduce animal stress if not implemented thoughtfully. Kling-Eveillard’s [112] research reveals that introducing PLF does not always degrade the human-animal relationship. Farmers implement new practices to familiarize animals with these technologies, demonstrating adaptability in the integration process. Additionally, farmers maintain agency in technology adoption, having room to maneuver when using tools or equipment and choosing to either entirely or partially delegate tasks to the equipment based on their judgment and experience. Han et al [8] suggest that integrating multiple parameters, such as pH, motility, and temperature, into comprehensive AI-driven DSS could improve data utility by prioritizing actionable alerts, but at which social price? Alarm fatigue and data overload present significant barriers to PLF adoption. The constant stream of sensor-generated alerts can overwhelm farm managers, leading to misinterpretation or desensitization of important alerts. This technological burden contributes to farmers’ expressed concerns about the potential loss of observation skills and developing dependence on PLF tools, highlighting the need for balanced implementation that preserves traditional husbandry expertise while embracing technological advancement.

### Opportunities

To address these challenges, future efforts should focus on developing cost-effective, user-friendly technologies that are adaptable to diverse environmental conditions; promoting data standardization; integrating PLF with AI technologies, such as LLM; and advancing hybrid modeling approaches. In the longer term, quantum computing may also offer transformative capabilities for modeling complex biological systems and accelerating AI computations, although practical agricultural applications are still emerging [113].

Challenges specific to precision nutrition modeling include the connectivity limitations common in rural farming operations. To mitigate these issues, satellite-based internet services and localized edge computing solutions have been proposed as alternative strategies to ensure real-time performance even in regions with poor connectivity. Edge computing offers promising solutions to these challenges, as demonstrated by Caria et al [85], who developed an intelligent pasture monitoring system that processes data locally using Raspberry Pi devices before transmitting summarized information when connectivity becomes available. This approach enables sophisticated nutrition modeling to function even in remote areas with limited internet access, which is crucial for widespread adoption.

The shift toward open-source platforms is emerging as a key area of innovation, enabling collaborative development and reducing the costs of proprietary AI-driven livestock monitoring solutions. This will be essential to ensure broad adoption by the agriculture sector, including livestock producers, particularly in developing regions where livestock production is integral to food security. Modular PLF designs could allow farmers to incrementally integrate AI-based monitoring tools, reducing financial strain while maximizing long-term benefits [114].

Developing standardized data collection and sharing protocols is crucial to improving interoperability and scalability in PLF systems. A lack of data standardization currently limits cross-platform integration, preventing large-scale AI models from learning effectively across multiple datasets. Although incipient and lacking wide support, some researchers emphasize the need for global regulatory frameworks that mandate common data structures for PLF devices, ensuring seamless communication between sensor networks, cloud platforms, and AI-driven analytics systems [115]. Thus, greater collaboration between researchers, technology developers, and producers will be essential to creating standardized datasets and training programs that address the unique challenges of on-farm applications.

The integration of LLM into PLF systems offers another avenue for innovation. LLM could serve as virtual assistants by synthesizing weather data, sensor metrics, and predictive models to recommend optimal grazing schedules or detect early disease outbreaks, significantly reducing producers’ cognitive load and enhancing real-time decision-making accuracy. Cui et al [116] propose that LLM could integrate weather forecasting, sensor-derived health data, and AI-based behavioral analysis to optimize grazing schedules, disease detection, and feed formulations. LLM integrated with IoT-based livestock sensors can identify subtle physiological changes that precede disease outbreaks, reducing reliance on antibiotics and enhancing overall herd health [117].

In precision nutrition specifically, emerging autonomous feeding systems represent a significant opportunity for increasing efficiency while reducing environmental impact. These advanced nutrition models can incorporate environmental footprint calculations alongside production metrics, enabling decision-making that optimizes economic and ecological outcomes. Producers can create comprehensive management platforms by integrating nutrition models with other PLF systems that dynamically adjust feeding strategies based on real-time health, production, and environmental data. A critical research priority in PLF is developing AI-driven sustainability models integrating satellite-based remote sensing with on-ground livestock monitoring systems [31]. These hybrid AI models could enable dynamic grazing strategies that respond to seasonal variability, drought conditions, and climate-related shifts in pasture availability. Furthermore, AI-based predictive weather models could enhance forage availability mapping, enabling adaptive grazing strategies that reduce environmental degradation and increase pasture efficiency [118].

### Outlook

The evolution of PLF technologies has not been without challenges. Additionally, interoperability between different devices and data formats (lack of standardized protocols) remains a significant barrier to widespread adoption. Future research should focus on developing universal data-sharing standards to facilitate interoperability between IoT devices, cloud platforms, and AI algorithms [80]. Furthermore, technological innovations must balance performance with accessibility, cost-effectiveness, and user-friendliness to accelerate adoption across diverse farming operations.

The future of precision nutrition modeling will likely involve increasingly autonomous systems that can assess animal status, predict responses to dietary changes, and implement optimal feeding strategies with minimal human intervention. Babina et al [119] reported a doubling of AI-related positions in agriculture and livestock between 2015 and 2018 compared to the preceding seven years in the United States, reflecting growing investment in automated systems powered by AI. Their research demonstrated economic and nutritional benefits from these self-optimizing systems, particularly in operations with frequent ingredient changes or variable animal requirements.

The successful integration of AI and digital technologies into livestock farming requires interdisciplinary collaboration among animal scientists, engineers, computer scientists, and social scientists. PLF adoption is not solely a technological challenge but also a socioeconomic and behavioral one, requiring educational programs, policy support, and farmer engagement and training [120]. Future PLF development should focus on creating systems that complement rather than replace traditional husbandry knowledge. The ideal trajectory would involve technologies that enhance farmers’ capabilities while preserving their autonomy and expertise, bridging the gap between innovation and practical on-farm implementation. For PLF to reach its full potential, the field must address disparities in access to technology and ensure that innovations benefit producers of all scales. This will require coordinated efforts from technology developers, policymakers, and educational institutions to create accessible, adaptable solutions that work across diverse farming contexts. As PLF technologies mature, developing appropriate regulatory frameworks will become increasingly important. These frameworks should balance innovation with ethical considerations, ensuring that technological advancement respects animal welfare, environmental sustainability, and farmers’ rights.

As precision nutrition modeling continues to evolve, the convergence of biological understanding, computational capabilities, and on-farm practicality will determine the pace and extent of adoption. Integrating PLF technologies with nutrition models represents a technological advancement and a fundamental shift in livestock management philosophy—from reactive, population-based approaches to proactive, individualized care. This paradigm shift promises to simultaneously address the seemingly competing goals of enhanced productivity, improved animal welfare, and reduced environmental impact. Realizing this potential will require continued interdisciplinary collaboration between animal scientists, data scientists, engineers, and, perhaps most importantly, the livestock producers who will ultimately implement these technologies in daily practice.

## CONCLUSION

PLF has evolved rapidly since 2017, moving from basic sensors to advanced AI-driven DSS. These technologies offer powerful tools to improve livestock sustainability, efficiency, and welfare. Progress in CV, AI, and the IoT has created new possibilities for animal monitoring, nutrition, and health management. However, important challenges remain. These include ensuring data privacy and security, reducing costs for small-scale producers, and improving the adaptability of AI models to different farm conditions. Climate change will further drive the need for resilient PLF systems that reduce environmental impact. A major advancement is the development of hybrid intelligent models that combine biological knowledge with ML. These systems can adjust feeding in real-time, improving resource use and sustainability. To support widespread adoption, future PLF tools must be easy to use, transparent, and adaptable to varying farm sizes and regions. Involving farmers in technology development and using multi-sensor approaches will be key to ensuring practical solutions. Ultimately, integrating biology with technology will help create livestock systems that are both precise and sustainable.

## Figures and Tables

**Table 1:** Summary table of artificial intelligence (AI) applications and key technologies utilized in cattle production

AI application	Key technologies	Purposes	References
CV in livestock	CNN (YOLO, ResNet), biometric facial recognition, IoT-Based Monitoring	Automates tracking of feeding behavior, weight estimation, and disease detection	[6,121,122]
AI and hybrid models for predictive analytics	Hybrid AI+mechanistic models, predictive AI for feed optimization, LSTM for disease detection	Improvement of feed efficiency, prediction of disease onset, and reduction of nutrient wastes	[93,123]
AI for livestock behavior analysis	Attention-based deep learning, CNN+transformer models for behavior tracking	Enhancement of stress detection, monitoring social interactions, and predicting behavioral patterns	[124–126]
AI in reproduction and precision breeding	RNN-based estrus detection, AI+accelerometer data for reproductive management	Increase accuracy in estrus detection, improve calving intervals, and enhance breeding success	[127,128]
AI for sustainable and autonomous PLF	AI-driven DSS, AI-powered drones for free-range livestock monitoring	Reduction of labor costs, optimization of resource allocation, and enabling autonomous farm management	[129]

CV, computer vision; CNN, convolutional neural network; IoT, internet of things; LSTM, long short-term memory; RNN, recurrent neural network; PLF, precision livestock farming; DSS, decision support systems.

## Data Availability

Upon reasonable request, the datasets of this study can be available from the corresponding author.

## References

[1] Berckmans D Automatic on-line monitoring of animals by precision livestock farming Geers R Madec F Livestock production and society International Society for Animal Hygiene (ISAH) 2004 27 30

[2] Berckmans D General introduction to precision livestock farming Anim Front 2017 7 6 11 10.2527/af.2017.0102

[3] Halachmi I Guarino M Editorial: precision livestock farming: a ‘per animal’ approach using advanced monitoring technologies Animal 2016 10 1482 3 10.1017/S1751731116001142 27534883

[4] Kleen JL Guatteo R Precision livestock farming: what does it contain and what are the perspectives? Animals 2023 13 779 10.3390/ani13050779 36899636 PMC10000125

[5] Berckmans D Advances in precision livestock farming Burleigh Dodds Science Publishing 2021

[6] Guarnido-Lopez P Pi Y Tao J Mendes EDM Tedeschi LO Computer vision algorithms to help decision-making in cattle production Anim Front 2024 14 11 22 10.1093/af/vfae028 39764526 PMC11700597

[7] González LA Kyriazakis I Tedeschi LO Review: precision nutrition of ruminants: approaches, challenges and potential gains Animal 2018 12 s246 61 10.1017/S1751731118002288 30277175

[8] Han CS Kaur U Bai H Invited review: sensor technologies for real-time monitoring of the rumen environment J Dairy Sci 2022 105 6379 404 10.3168/jds.2021-20576 35773034

[9] Romano E Brambilla M Cutini M Increased cattle feeding precision from automatic feeding systems: considerations on technology spread and farm level perceived advantages in Italy Animals 2023 13 3382 10.3390/ani13213382 37958137 PMC10649016

[10] Tedeschi LO Fonseca MA Muir JP A glimpse of the future in animal nutrition science. 2. Current and future solutions Rev Bras Zootec 2017 46 452 69 10.1590/s1806-92902017000500012

[11] Saar M Edan Y Godo A Lepar J Parmet Y Halachmi I A machine vision system to predict individual cow feed intake of different feeds in a cowshed Animal 2022 16 100432 10.1016/j.animal.2021.100432 35007881

[12] Wang S Jiang H Qiao Y Jiang S Lin H Sun Q The research progress of vision-based artificial intelligence in smart pig farming Sensors 2022 22 6541 10.3390/s22176541 36080994 PMC9460267

[13] Tedeschi LO Muir JP Riley DG Fox DG The role of ruminant animals in sustainable livestock intensification programs Int J Sustain Dev World Ecol 2015 22 452 65 10.1080/13504509.2015.1075441

[14] Morrone S Dimauro C Gambella F Cappai MG Industry 4.0 and precision livestock farming (PLF): an up to date overview across animal productions Sensors 2022 22 4319 10.3390/s22124319 35746102 PMC9228240

[15] Lamanna M Bovo M Cavallini D Wearable collar technologies for dairy cows: a systematized review of the current applications and future innovations in precision livestock farming Animals 2025 15 458 10.3390/ani15030458 39943229 PMC11815998

[16] Tekin K Yurdakök Dikmen B Kanca H Guatteo R Precision livestock farming technologies: novel direction of information flow Ankara Üniv Vet Fak Derg 2021 68 193 212 10.33988/auvfd.837485

[17] Abi-Rizk H Jouan-Rimbaud Bouveresse D Chamberland J Cordella CBY Recent developments of e-sensing devices coupled to data processing techniques in food quality evaluation: a critical review Anal Methods 2023 15 5410 40 10.1039/D3AY01132A 37818969

[18] Dijkstra J van Gastelen S Dieho K Nichols K Bannink A Review: rumen sensors: data and interpretation for key rumen metabolic processes Animal 2020 14 s176 86 10.1017/S1751731119003112 32024561

[19] Wu D Yin X Jiang B Jiang M Li Z Song H Detection of the respiratory rate of standing cows by combining the Deeplab V3+ semantic segmentation model with the phase-based video magnification algorithm Biosyst Eng 2020 192 72 89 10.1016/j.biosystemseng.2020.01.012

[20] Yaseer A Chen H A review of sensors and machine learning in animal farming Proceedings of the 2021 IEEE 11th Annual International Conference on CYBER Technology in Automation, Control, and Intelligent Systems (CYBER) 2021 Jul 27–31 Jiaxing, China IEEE 2021 747 52

[21] Qiao Y Guo Y He D Cattle body detection based on YOLOv5-ASFF for precision livestock farming Comput Electron Agric 2023 204 107579 10.1016/j.compag.2022.107579

[22] Korkmaz A Agdas MT Kosunalp S Iliev T Stoyanov I Detection of threats to farm animals using deep learning models: a comparative study Appl Sci 2024 14 6098 10.3390/app14146098

[23] Rohan A Rafaq MS Hasan MJ Asghar F Bashir AK Dottorini T Application of deep learning for livestock behaviour recognition: a systematic literature review Comput Electron Agric 2024 224 109115 10.1016/j.compag.2024.109115

[24] Ramirez BC Xin H Halbur PG At the intersection of industry, academia, and government: how do we facilitate productive precision livestock farming in practice? Animals 2019 9 635 10.3390/ani9090635 31480220 PMC6770712

[25] Mallinger K Corpaci L Neubauer T Tikász IE Banhazi T Unsupervised and supervised machine learning approach to assess user readiness levels for precision livestock farming technology adoption in the pig and poultry industries Comput Electron Agric 2023 213 108239 10.1016/j.compag.2023.108239

[26] Silvi R Pereira LGR Paiva CAV Adoption of precision technologies by Brazilian dairy farms: the farmer’s perception Animals 2021 11 3488 10.3390/ani11123488 34944264 PMC8698152

[27] Tedeschi LO Mendes EDM 13 Precision livestock farming tools for climate-smart feedyard operations J Anim Sci 2023 101 326 7 10.1093/jas/skad281.390

[28] Adams JM Fernandes J Jr Fernandes M Reis R Tedeschi LO PSII-19 a satellite-based decision-support tool to optimize profitability and environmental stewardship of cow-calf operations J Anim Sci 2023 101 481 10.1093/jas/skad281.571

[29] Fernandes MH Fernandes J Reis RA Tedeschi LO PSXII-9 application of machine learning algorithms to estimate tropical pasture biomass based on satellite images J Anim Sci 2022 100 284 5 10.1093/jas/skac247.518

[30] Fernandes MHMR Tedeschi LO 2 Satellite-based decision support tools to assist grazing cattle production J Anim Sci 2023 101 74 5 10.1093/jas/skad281.091

[31] Fernandes MHMR de Souza Fernandes J Junior Adams JM Lee M Reis RA Tedeschi LO Using sentinel-2 satellite images and machine learning algorithms to predict tropical pasture forage mass, crude protein, and fiber content Sci Rep 2024 14 8704 10.1038/s41598-024-59160-x 38622291 PMC11018762

[32] Fernandes MHMR Tedeschi LO ASAS-NANP symposim: mathematical moedling in animal nutrition: application of modeling innovations to support satellite remote sensing for sustainable grazing cattle management J Anim Sci 2025 103 skaf137 10.1093/jas/skaf137 40319360 PMC12351259

[33] Kamilaris A Prenafeta-Boldú FX A review of the use of convolutional neural networks in agriculture J Agric Sci 2018 156 312 22 10.1017/S0021859618000436

[34] Meng H Zhang L Yang F Livestock biometrics identification using computer vision approaches: a review Agriculture 2025 15 102 10.3390/agriculture15010102

[35] Hostiou N Fagon J Chauvat S Impact of precision livestock farming on work and human-animal interactions on dairy farms. A review Biotechnol Agron Soc Environ 2017 21 268 75 10.25518/1780-4507.13706

[36] Bezen R Edan Y Halachmi I Computer vision system for measuring individual cow feed intake using RGB-D camera and deep learning algorithms Comput Electron Agric 2020 172 105345 10.1016/j.compag.2020.105345

[37] Guarnido Lopez PP Ramirez-Agudelo F Ribeira-Ferreiro E Aouda MB 301 Determining individual dry matter intake of Charolais bulls from ingestion time recorded with Rgb-D cameras J Anim Sci 2023 101 247 8 10.1093/jas/skad281.297

[38] Lowe G Sutherland M Waas J Schaefer A Cox N Stewart M Infrared thermography: a non-invasive method of measuring respiration rate in calves Animals 2019 9 535 10.3390/ani9080535 31394802 PMC6720651

[39] Davison C Bowen JM Michie C Predicting feed intake using modelling based on feeding behaviour in finishing beef steers Animal 2021 15 100231 10.1016/j.animal.2021.100231 34116464 PMC8282503

[40] Shangru L Chengrui Z Ruixue W Establishment of a feed intake prediction model based on eating time, ruminating time and dietary composition Comput Electron Agric 2022 202 107296 10.1016/j.compag.2022.107296

[41] Ding Y Hua L Li S Research on computer vision enhancement in intelligent robot based on machine learning and deep learning Neural Comput Appl 2022 34 2623 35 10.1007/s00521-021-05898-8

[42] Nasirahmadi A Edwards SA Sturm B Implementation of machine vision for detecting behaviour of cattle and pigs Livest Sci 2017 202 25 38 10.1016/j.livsci.2017.05.014

[43] Rahman A Smith DV Little B Ingham AB Greenwood PL Bishop-Hurley GJ Cattle behaviour classification from collar, halter, and ear tag sensors Inf Process Agric 2018 5 124 33 10.1016/j.inpa.2017.10.001

[44] Gebreyesus G Milkevych V Lassen J Sahana G Supervised learning techniques for dairy cattle body weight prediction from 3D digital images Front Genet 2023 13 947176 10.3389/fgene.2022.947176 36685975 PMC9849234

[45] Wang J Dai B Li Y He Y Sun Y Shen W An intelligent edge-IoT platform with deep learning for body condition scoring of dairy cow IEEE Internet Things J 2024 11 17453 67 10.1109/JIOT.2024.3357862

[46] Gjergji M Weber VM Silva LOC Deep learning techniques for beef cattle body weight prediction Proceedings of the 2020 International Joint Conference on Neural Networks (IJCNN) 2020 Jul 19–24 Glasgow, UK IEEE 2020 1 8

[47] Ji W Luo Y Liao Y UAV assisted livestock distribution monitoring and quantification: a low-cost and high-precision solution Animals 2023 13 3069 10.3390/ani13193069 37835675 PMC10571782

[48] Liu H Reibman AR Boerman JP Feature extraction using multi-view video analytics for dairy cattle body weight estimation Smart Agric Technol 2023 6 100359 10.1016/j.atech.2023.100359

[49] Xu B Wang W Falzon G Automated cattle counting using Mask R-CNN in quadcopter vision system Comput Electron Agric 2020 171 105300 10.1016/j.compag.2020.105300

[50] Crowe MA Hostens M Opsomer G Reproductive management in dairy cows - the future Ir Vet J 2018 71 1 10.1186/s13620-017-0112-y PMC5759237 29321918

[51] Das S Shaji A Nain D Singha S Karunakaran M Baithalu RK Precision technologies for the management of reproduction in dairy cows Trop Anim Health Prod 2023 55 286 10.1007/s11250-023-03704-2 37540276

[52] Roche JF Mackey D Diskin MD Reproductive management of postpartum cows Anim Prod Sci 2000 60–61 703 12 10.1016/S0378-4320(00)00107-X 10844236

[53] Fricke PM Carvalho PD Giordano JO Valenza A Lopes G Jr Amundson MC Expression and detection of estrus in dairy cows: the role of new technologies Animal 2014 8 134 43 10.1017/S1751731114000299 24680286

[54] Valenza A Giordano JO Lopes G Jr Vincenti L Amundson MC Fricke PM Assessment of an accelerometer system for detection of estrus and treatment with gonadotropin-releasing hormone at the time of insemination in lactating dairy cows J Dairy Sci 2012 95 7115 27 10.3168/jds.2012-5639 23040033

[55] Madureira AML Silper BF Burnett TA Factors affecting expression of estrus measured by activity monitors and conception risk of lactating dairy cows J Dairy Sci 2015 98 7003 14 10.3168/jds.2015-9672 26254517

[56] Saint-Dizier M Chastant-Maillard S Methods and on-farm devices to predict calving time in cattle Vet J 2015 205 349 56 10.1016/j.tvjl.2015.05.006 26164528

[57] Reith S Hoy S Relationship between daily rumination time and estrus of dairy cows J Dairy Sci 2012 95 6416 20 10.3168/jds.2012-5316 22939783

[58] Pahl C Hartung E Mahlkow-Nerge K Haeussermann A Feeding characteristics and rumination time of dairy cows around estrus J Dairy Sci 2015 98 148 54 10.3168/jds.2014-8025 25465539

[59] Brehme U Stollberg U Holz R Schleusener T ALT pedometer: new sensor-aided measurement system for improvement in oestrus detection Comput Electron Agric 2008 62 73 80 10.1016/j.compag.2007.08.014

[60] Jung DH Kim NY Moon SH Deep learning-based cattle vocal classification model and real-time livestock monitoring system with noise filtering Animals 2021 11 357 10.3390/ani11020357 33535390 PMC7911430

[61] Sharma S Kadyan V Detection of estrus through automated classification approaches using vocalization pattern in Murrah buffaloes Proceedings of the 2023 3rd International Conference on Artificial Intelligence and Signal Processing (AISP) 2023 Mar 18–20 Vijayawada, India IEEE 2023 1 6

[62] Wang Y Kang X He Z Feng Y Liu G Accurate detection of dairy cow mastitis with deep learning technology: a new and comprehensive detection method based on infrared thermal images animal 2022 16 100646 10.1016/j.animal.2022.100646 36183435

[63] Sejian V Devaraj C Shashank CG Heat stress in ruminants and its mitigation through nutritional additives Mahesh MS Yata VK Feed additives and supplements for ruminants Springer Nature 2024 367 98

[64] Michelena Á Aveleira-Mata J Jove E A novel intelligent approach for man-in-the-middle attacks detection over Internet of things environments based on message queuing telemetry transport Expert Syst 2024 41 e13263 10.1111/exsy.13263

[65] Mylostyvyi R Sejian V Souza-Junior JBF Digitalisation opportunities for livestock welfare monitoring with a focus on heat stress Multidiscip Rev 2024 7 2024300 10.31893/multirev.2024300

[66] Rebez EB Sejian V Silpa MV Applications of artificial intelligence for heat stress management in ruminant livestock Sensors 2024 24 5890 10.3390/s24185890 39338635 PMC11435989

[67] Ghassemi Nejad J Ju MS Jo JH Advances in methane emission estimation in livestock: a review of data collection methods, model development and the role of AI technologies Animals 2024 14 435 10.3390/ani14030435 38338080 PMC10854801

[68] Kasimanickam R Ferreira JCP Kastelic J Kasimanickam V Application of genomic selection in beef cattle disease prevention Animals 2025 15 277 10.3390/ani15020277 39858277 PMC11759163

[69] Hajnal É Kovács L Vakulya G Dairy cattle rumen bolus developments with special regard to the applicable artificial intelligence (AI) methods Sensors 2022 22 6812 10.3390/s22186812 36146158 PMC9505622

[70] Vargas-Bello-Pérez E Espinoza-Sandoval OR Gonzalez Ronquillo M The role of artificial intelligence in Latin American ruminant production systems Anim Front 2024 14 23 32 10.1093/af/vfae034 39764528 PMC11700588

[71] Phillips N Mottram T Poppi D Mayer D McGowan MR Continuous monitoring of ruminal pH using wireless telemetry Anim Prod Sci 2009 50 72 7 10.1071/AN09027

[72] Campuzano S Pedrero M Yáñez-Sedeño P Pingarrón JM Antifouling (bio)materials for electrochemical (bio)sensing Int J Mol Sci 2019 20 423 10.3390/ijms20020423 30669466 PMC6358752

[73] Neubauer V Humer E Kröger I Braid T Wagner M Zebeli Q Differences between pH of indwelling sensors and the pH of fluid and solid phase in the rumen of dairy cows fed varying concentrate levels J Anim Physiol Anim Nutr 2018 102 343 9 10.1111/jpn.12675 28111836

[74] Crossland WL Cagle CM Sawyer JE Callaway TR Tedeschi LO Evaluation of active dried yeast in the diets of feedlot steers. II. Effects on rumen pH and liver health of feedlot steers J Anim Sci 2019 97 1347 63 10.1093/jas/skz008 30753501 PMC6396254

[75] Crossland WL Jobe JT Ribeiro FRB Sawyer JE Callaway TR Tedeschi LO Evaluation of active dried yeast in the diets of feedlot steers—I: effects on feeding performance traits, the composition of growth, and carcass characteristics J Anim Sci 2019 97 1335 46 10.1093/jas/skz007 30657913 PMC6396235

[76] Cusack PMV Dell’Osa D Wilkes G Grandini D Tedeschi LO Ruminal pH and its relationship with dry matter intake, growth rate, and feed conversion ratio in commercial Australian feedlot cattle fed for 148 days Aust Vet J 2021 99 319 25 10.1111/avj.13069 33851419

[77] Dias Batista LF Rivera ME Tedeschi LO Investigation of virginiamycin to improve health of growing and finishing steers: II. animal growth and development, and intake dynamics Appl Anim Sci 2024 40 487 503 10.15232/aas.2023-02510

[78] Rivera ME Dias Batista LF Tedeschi LO Investigation of virginiamycin to improve health of growing and finishing steers: I. effects on ruminal acidosis and liver health Appl Anim Sci 2024 40 329 46 10.15232/aas.2023-02511

[79] Tarafdar A Sheikh A Majumder P Enhancing intrusion detection using wireless sensor networks: a novel ahp-madm aggregated multiple type 3 fuzzy logic-based k-barriers prediction system Peer Peer Netw Appl 2024 17 1732 49 10.1007/s12083-024-01688-w

[80] Tedeschi LO Greenwood PL Halachmi I Advancements in sensor technology and decision support intelligent tools to assist smart livestock farming J Anim Sci 2021 99 1 11 10.1093/jas/skab038 PMC7896629 33550395

[81] Gupta S Nirmala TV Reddy AD Kumar D Smart livestock farming: monitoring and health management through IoT Gupta S Hasan W Singh S Kumar D Ansari MJ Nisar S Agriculture 4.0 CRC Press 2024

[82] Fuentes-Peñailillo F Gutter K Vega R Silva GC Transformative technologies in digital agriculture: leveraging Internet of things, remote sensing, and artificial intelligence for smart crop management J Sens Actuator Netw 2024 13 39 10.3390/jsan13040039

[83] Vlaicu PA Gras MA Untea AE Lefter NA Rotar MC Advancing livestock technology: intelligent systemization for enhanced productivity, welfare, and sustainability AgriEngineering 2024 6 1479 96 10.3390/agriengineering6020084

[84] Alonso RS Sittón-Candanedo I García Ó Prieto J Rodríguez-González S An intelligent edge-IoT platform for monitoring livestock and crops in a dairy farming scenario Ad Hoc Netw 2020 98 102047 10.1016/j.adhoc.2019.102047

[85] Caria M Schudrowitz J Jukan A Kemper N Smart farm computing systems for animal welfare monitoring Proceedings of the 2017 40th International Convention on Information and Communication Technology, Electronics and Microelectronics (MIPRO) 2017 May 22–26 Opatija, Croatia IEEE 2017 152 7

[86] National Academies of Sciences, Engineering, and Medicine Nutrient requirements of beef cattle 8th ed The National Academies Press 2016

[87] National Academies of Sciences, Engineering, and Medicine Nutrient requirements of dairy cattle 8th ed The National Academies Press 2021 38386771

[88] Tedeschi LO Fox DG The ruminant nutrition system: an applied model for predicting nutrient requirements and feed utilization in ruminants 3rd ed Kendall Hunt 2020

[89] Tedeschi LO Fox DG The ruminant nutrition system: tables of equations and coding 1st ed Kendall Hunt 2020

[90] Pomar C Pomar J Babot D Dubeau F Effet d’une alimentation multiphase quotidienne sur les performances zootechniques, la composition corporelle et les rejets d’azote et de phosphore du porc charcutier J Recherche Porcine 2007 39 23 30

[91] Pomar C Pomar J Dubeau F Joannopoulos E Dussault JP The impact of daily multiphase feeding on animal performance, body composition, nitrogen and phosphorus excretions, and feed costs in growing–finishing pigs Animal 2014 8 704 13 10.1017/S1751731114000408 24739349

[92] Awasthi TR Morshed A Swain DL A machine learning approach to simulate cattle growth at pasture using remotely collected walk-over weights Agric Syst 2025 226 104332 10.1016/j.agsy.2025.104332

[93] Tedeschi LO Review: the prevailing mathematical modeling classifications and paradigms to support the advancement of sustainable animal production animal 2023 17 100813 10.1016/j.animal.2023.100813 37169649

[94] Allen MS Do more mechanistic models increase accuracy of prediction of metabolisable protein supply in ruminants? Anim Prod Sci 2019 59 1991 8 10.1071/AN19337

[95] Neethirajan S Kemp B Digital twins in livestock farming Animals [Preprint] 2021 [cited 2025 Jan 1]. 10.20944/preprints202101.0620.v1 PMC8065673 33916713

[96] Neethirajan S Twin farms nexus: digital twins for sustainable animal farming Arch Anim Poult Sci 2024 2 555595 10.19080/AAPS.2024.02.555595

[97] Tedeschi LO ASAS-NANP symposium: mathematical modeling in animal nutrition: the progression of data analytics and artificial intelligence in support of sustainable development in animal science J Anim Sci 2022 100 1 11 10.1093/jas/skac111 PMC9171329 35412610

[98] Monteiro HF Figueiredo CC Mion B An artificial intelligence approach of feature engineering and ensemble methods depicts the rumen microbiome contribution to feed efficiency in dairy cows Anim Microbiome 2024 6 5 10.1186/s42523-024-00289-5 38321581 PMC10845535

[99] Fontanesi L Metabolomics and livestock genomics: insights into a phenotyping frontier and its applications in animal breeding Anim Front 2016 6 73 9 10.2527/af.2016-0011

[100] de Novais FJ Yu H Cesar ASM Multi-omic data integration for the study of production, carcass, and meat quality traits in Nellore cattle Front Genet 2022 13 948240 10.3389/fgene.2022.948240 36338989 PMC9634488

[101] Lee M Kim DH Seo S Tedeschi LO Development of machine learning models for estimating metabolizable protein supply from feed in lactating dairy cows Animals 2025 15 687 10.3390/ani15050687 40075970 PMC11898976

[102] Eastwood CR Chapman DF Paine MS Networks of practice for co-construction of agricultural decision support systems: case studies of precision dairy farms in Australia Agric Syst 2012 108 10 8 10.1016/j.agsy.2011.12.005

[103] Groenendaal H Galligan DT Mulder HA An economic spreadsheet model to determine optimal breeding and replacement decisions for dairy cattle J Dairy Sci 2004 87 2146 57 10.3168/jds.S0022-0302(04)70034-X 15328228

[104] Wolfert S Ge L Verdouw C Bogaardt MJ Big data in smart farming: a review Agric Syst 2017 153 69 80 10.1016/j.agsy.2017.01.023

[105] Zuidhof MJ Precision livestock feeding: matching nutrient supply with nutrient requirements of individual animals J Appl Poult Res 2020 29 11 4 10.1016/j.japr.2019.12.009

[106] Ghazal S Munir A Qureshi WS Computer vision in smart agriculture and precision farming: techniques and applications Artif Intell Agric 2024 13 64 83 10.1016/j.aiia.2024.06.004

[107] Nyamuryekung’e S Transforming ranching: precision livestock management in the Internet of things era Rangelands 2024 46 13 22 10.1016/j.rala.2023.10.002

[108] Jiang B Tang W Cui L Deng X Precision livestock farming research: a global scientometric review Animals 2023 13 2096 10.3390/ani13132096 37443894 PMC10340063

[109] Balkrishna A Pathak R Kumar S Arya V Singh SK A comprehensive analysis of the advances in Indian digital agricultural architecture Smart Agric Technol 2023 5 100318 10.1016/j.atech.2023.100318

[110] Raihan A Ridwan M Rahman MS An exploration of the latest developments, obstacles, and potential future pathways for climate-smart agriculture Clim Smart Agric 2024 1 100020 10.1016/j.csag.2024.100020

[111] Pomar C Remus A Review: fundamentals, limitations and pitfalls on the development and application of precision nutrition techniques for precision livestock farming animal 2023 17 100763 10.1016/j.animal.2023.100763 36966025

[112] Kling-Eveillard F Allain C Boivin X Farmers’ representations of the effects of precision livestock farming on human-animal relationships Livest Sci 2020 238 104057 10.1016/j.livsci.2020.104057

[113] Tedeschi LO 6 Transforming animal agriculture through hybrid modeling and quantum computing J Anim Sci 2024 102 70 1 10.1093/jas/skae234.078

[114] Nastasijevic I Kundacina I Jaric S Pavlovic Z Radovic M Radonic V Recent advances in biosensor technologies for meat production chain Foods 2025 14 744 10.3390/foods14050744 40077447 PMC11899517

[115] Yang Y Lin M Lin Y Zhang C Wu C A survey of blockchain applications for management in agriculture and livestock Internet of things Future Internet 2025 17 40 10.3390/fi17010040

[116] Cui S Lyu S Ma Y Wang K Improved informer PV power short-term prediction model based on weather typing and AHA-VMD-MPE Energy 2024 307 132766 10.1016/j.energy.2024.132766

[117] Gupta GK Singh A Manikandan SV Ehtesham A Digital diagnostics: the potential of large language models in recognizing symptoms of common illnesses AI 2025 6 13 10.3390/ai6010013

[118] Al Mamun MA Sarker MR Sarkar MAR Identification of influential weather parameters and seasonal drought prediction in Bangladesh using machine learning algorithm Sci Rep 2024 14 566 10.1038/s41598-023-51111-2 38177219 PMC10767098

[119] Babina T Fedyk A He A Hodson J Artificial intelligence, firm growth, and product innovation J Financ Econ 2024 151 103745 10.1016/j.jfineco.2023.103745

[120] de Oliveira FM Ferraz GAS André ALG Santana LS Norton T Ferraz PFP Digital and precision technologies in dairy cattle farming: a bibliometric analysis Animals 2024 14 1832 10.3390/ani14121832 38929450 PMC11201094

[121] Bao Y Llagostera P Plà-Aragonès LM Is deep learning useful for decision making in pig production? Internet Things 2024 26 101229 10.1016/j.iot.2024.101229

[122] Neethirajan S From predictive analytics to emotional recognition–the evolving landscape of cognitive computing in animal welfare Int J Cogn Comput Eng 2024 5 123 31 10.1016/j.ijcce.2024.02.003

[123] Wang H Liu J Dong Z Song J Zhu Z Artificial intelligence-based metabolic energy prediction model for animal feed proportioning optimization Ital J Anim Sci 2023 22 942 52 10.1080/1828051X.2023.2236132

[124] Gao G Wang C Wang J CNN-Bi-LSTM: a complex environment-oriented cattle behavior classification network based on the fusion of CNN and Bi-LSTM Sensors 2023 23 7714 10.3390/s23187714 37765771 PMC10536225

[125] Li G Shi G Zhu C Dynamic serpentine convolution with attention mechanism enhancement for beef cattle behavior recognition Animals 2024 14 466 10.3390/ani14030466 38338110 PMC10854982

[126] Qiao Y Guo Y Yu K He D C3D-ConvLSTM based cow behaviour classification using video data for precision livestock farming Comput Electron Agric 2022 193 106650 10.1016/j.compag.2021.106650

[127] Akinsulie OC Idris I Aliyu VA The potential application of artificial intelligence in veterinary clinical practice and biomedical research Front Vet Sci 2024 11 1347550 10.3389/fvets.2024.1347550 38356661 PMC10864457

[128] Hoorn QA Rabaglino MB Amaral TF Machine learning to identify endometrial biomarkers predictive of pregnancy success following artificial insemination in dairy cows Biol Reprod 2024 111 54 62 10.1093/biolre/ioae052 38590174

[129] Tzanidakis C Simitzis P Panagakis P Precision livestock farming (PLF) systems: improving sustainability and efficiency of animal production García Márquez FP Lev B Sustainability: cases and studies in using operations research and management science methods Springer 2023 285 337

[130] Tedeschi LO Quantifying ruminal health: a statistical review and application of area and time under the curve in animal science Ecol Inform 2025 90 103271 10.1016/j.ecoinf.2025.103271

[131] Tedeschi LO ASAS-NANP symposium: mathematical modeling in animal nutrition: synthetic database generation for non-normal multivariate distributions: a rank-based method with application to ruminant methane emissions J Anim Sci 2025 103 skaf136 10.1093/jas/skaf136 40319357 PMC12351256

